# Metabolism of Aldoximes and Nitriles in Plant-Associated Bacteria and Its Potential in Plant-Bacteria Interactions

**DOI:** 10.3390/microorganisms10030549

**Published:** 2022-03-02

**Authors:** Robert Rädisch, Miroslav Pátek, Barbora Křístková, Margit Winkler, Vladimír Křen, Ludmila Martínková

**Affiliations:** 1 Institute of Microbiology of the Czech Academy of Sciences, Vídeňská 1083, CZ-142 20 Prague, Czech Republic; robert.radisch@biomed.cas.cz (R.R.); patek@biomed.cas.cz (M.P.); barbora.kristkova@biomed.cas.cz (B.K.); kren@biomed.cas.cz (V.K.); 2 Department of Genetics and Microbiology, Faculty of Sciences, Charles University, Viničná 5, CZ-128 44 Prague, Czech Republic; 3 Faculty of Food and Biochemical Technology, University of Chemistry and Technology, Technická 5, CZ-166 28 Prague, Czech Republic; 4 Institute of Molecular Biotechnology, Faculty of Technical Chemistry, Chemical and Process Engineering, Biotechnology, Graz University of Technology, Petersgasse 14, A-8010 Graz, Austria; margit.winkler@tugraz.at; 5 Austrian Center of Industrial Biotechnology GmbH, Krenngasse 37, A-8010 Graz, Austria

**Keywords:** plant aldoxime, aldoxime-nitrile pathway, plant defense, phytohormone, indole-3-acetic acid, aldoxime dehydratase, nitrilase, nitrile hydratase, plant-bacteria interaction

## Abstract

In plants, aldoximes per se act as defense compounds and are precursors of complex defense compounds such as cyanogenic glucosides and glucosinolates. Bacteria rarely produce aldoximes, but some are able to transform them by aldoxime dehydratase (Oxd), followed by nitrilase (NLase) or nitrile hydratase (NHase) catalyzed transformations. Oxds are often encoded together with NLases or NHases in a single operon, forming the aldoxime–nitrile pathway. Previous reviews have largely focused on the use of Oxds and NLases or NHases in organic synthesis. In contrast, the focus of this review is on the contribution of these enzymes to plant-bacteria interactions. Therefore, we summarize the substrate specificities of the enzymes for plant compounds. We also analyze the taxonomic and ecological distribution of the enzymes. In addition, we discuss their importance in selected plant symbionts. The data show that Oxds, NLases, and NHases are abundant in Actinobacteria and Proteobacteria. The enzymes seem to be important for breaking through plant defenses and utilizing oximes or nitriles as nutrients. They may also contribute, e.g., to the synthesis of the phytohormone indole-3-acetic acid. We conclude that the bacterial and plant metabolism of aldoximes and nitriles may interfere in several ways. However, further in vitro and in vivo studies are needed to better understand this underexplored aspect of plant-bacteria interactions.

## 1. Introduction

Oximes (RR′-C=N-OH) are metabolites that cross the boundaries between the kingdoms of life. They are widely distributed, especially in higher plants. Knowledge about plant oximes has been recently summarized [1]. Briefly, the plant oximes are largely aldoximes (R-CH=N-OH) or their methylated analogs (R-CH=N-OMe). They are derived from a few amino acids (Val, Leu, Ile, Met, Trp, Phe, and Tyr) and analogs of some of these acids. Aldoximes are intermediates in the biosynthesis of defense compounds (cyanogenic glucosides (CGs), glucosinolates (GSLs), and camalexin), but also per se have some biological activities. The functions of plant oximes are numerous, but not all of them are well understood. They include defense against pathogens and herbivores, growth control, radical scavenging, interaction with pollinators, interspecies communication, and nitrogen supply [1,2].

**Figure 1:**
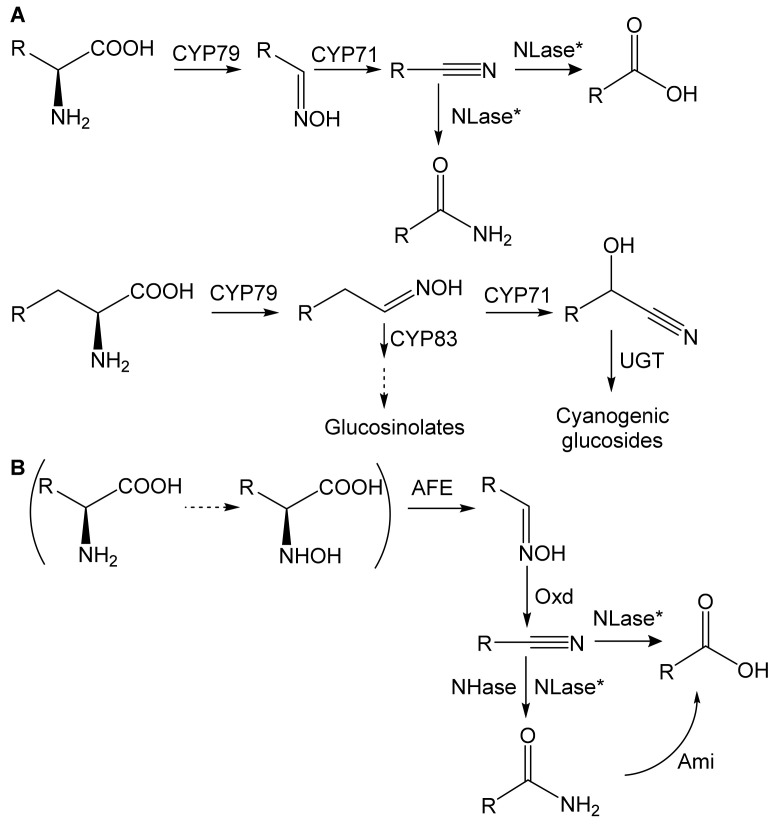
Metabolism of aldoximes in (**A**) plants and (**B**) bacteria (aldoxime-nitrile pathway). AFE, aldoxime-forming enzyme; Ami, amidase; CYP, cytochrome P450 (CYP71, CYP79, and CYP83 family); NHase, nitrile hydratase; NLase, nitrilase; Oxd, aldoxime dehydratase; UGT, UDP-glucosyl transferase; according to [1,4]. *NLase has two activities: acid- and amide-forming. The latter is usually minor. The pathway in brackets holds for R = CH_2_Ph. The dashed arrow means a hypothetical reaction.

In plants, the enzymes of oxime metabolism mainly belong to the cytochrome P450 (CYP) superfamily (Figure 1A); CYP79, CYP71, and CYP83 catalyze the synthesis of aldoximes, nitriles, and precursors of GSLs, respectively [1]. Simple nitriles (R-C≡N) are hydrolyzed by nitrilase (EC 3.5.5.-; NLase) [3], and hydroxynitriles (R-C(OH)C≡N) are precursors of CGs [1].

Oximes are rarely produced in bacteria. An exception is *Bacillus* sp. OxB-1, which forms phenylacetaldoxime (PAOx) from *N*-hydroxy-l-phenylalanine [4]. The latter is likely a metabolite of l-Phe but the enzyme that catalyzes the conversion of l-Phe is not known. The same strain is able to metabolize PAOx and other oximes (see below).

Bacterial transformation of oximes occurs via the aldoxime-nitrile pathway (ANP) (Figure 1B). Dehydration of an aldoxime to a nitrile is catalyzed by aldoxime dehydratase (Oxd; EC 4.99.1.-) [5]. Nitrile hydrolysis proceeds via two alternative pathways catalyzed by (i) NLase or (ii) nitrile hydratase (EC 4.2.1.84; NHase) and amidase (EC 3.5.1.4). Thus, the transformation of oximes in plants and bacteria involves the same metabolites (nitriles, carboxylic acids).

The industrial potential of the above enzymes has been summarized many times (e.g., [6,7,8,9]) but reviews on their natural roles are scarce. The in vivo functions of NLases were discussed by Piotrowski [3] and Howden and Preston [2], with the former work focusing on plant NLases. Aldoxime metabolism was briefly tackled in these works.

The metabolism of the phytohormone indole-3-acetic acid (IAA), which, under certain conditions, proceeds via indole-3-acetaldoxime (IAOx) or indole-3-acetonitrile (IAN), was reviewed previously [10]. However, the focus has been on other pathways to IAA that are more common (Section 4). The role of IAA in plant-bacteria interactions was also discussed in the study [10] and another review [11].

The above-mentioned review of natural, mainly plant oximes focused on the structures, distribution, biosynthesis, and biological activities of these compounds [1].

However, a review focusing on the role of Oxds and nitrile-converting enzymes in plant-bacteria interactions is lacking. The metabolism of oximes and nitriles is evidently important for plant-bacterial cross-talk, resulting in metabolites found in both microbes and plants. Therefore, in this survey, we will summarize what is known about the activities of bacterial Oxds, NLases, and NHases toward plant compounds.

We will also analyze the taxonomic and ecological distribution of these enzymes. In addition, we will discuss their role in some plant-growth-promoting bacteria and phytopathogens. Finally, we propose possible topics for future studies in this field.

## 2. Substrate Specificity

The transformation of the aldoxime IAOx into the corresponding nitrile in microbes (fungi) was noticed in the 1960s [12]. However, the discovery of the first Oxds was reported only about two decades ago. These were Oxds from *Rhodococcus* sp. YH3-3 [13], *Bacillus* sp. OxB-1 [14], and *Pseudomonas chlororaphis* B23 [15]. NLases have been known since the 1960s and their spectrum has been expanded since then [6,16]. The first report on NHase appeared in 1980 [17]. Previous reviews largely focused on the use of the enzymes for industrially important reactions [5,7,9]. In the following, we analyze the data on the substrate specificity of these enzymes for plant oximes and nitriles.

### 2.1. Aldoxime Dehydratases

**Figure 2:**
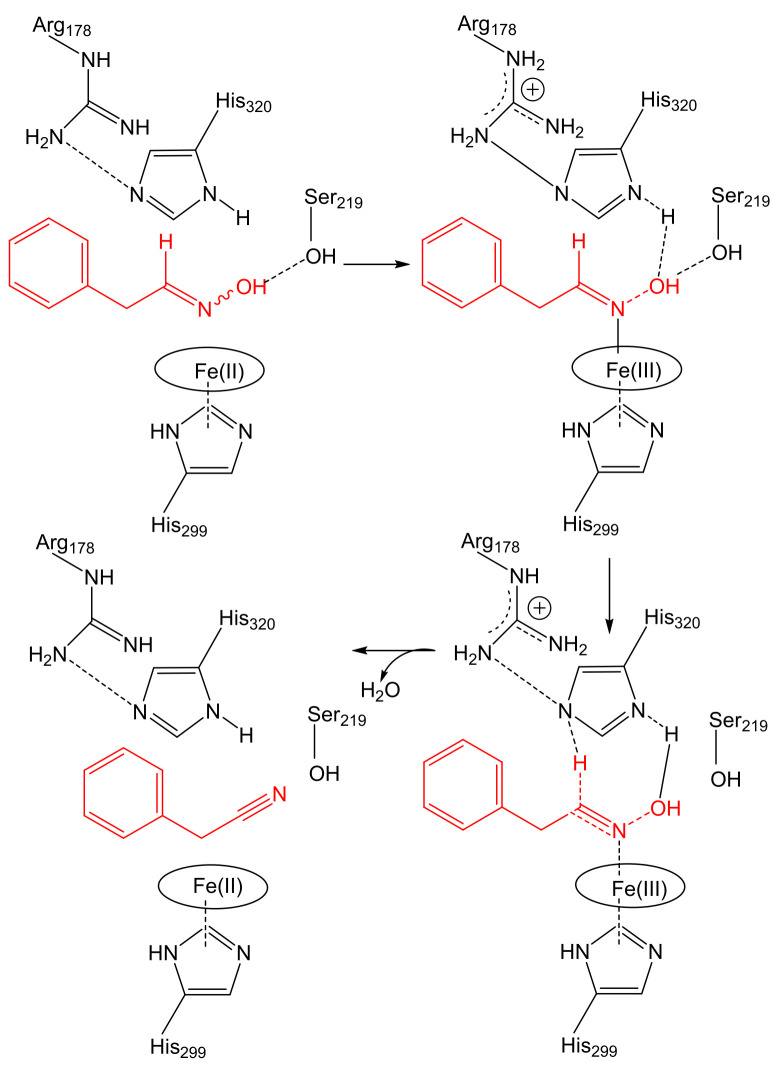
The binding of the substrate, phenylacetaldoxime, in the catalytic center of aldoxime dehydratase and release of the product, phenylacetonitrile; according to [18], modified. Amino acid numbering as in OxdA from *Pseudomonas chlororaphis* B23. Substrate and product in red.

Oxds are unique heme enzymes that catalyze the dehydration of aldoximes in an aqueous environment. The catalytic Fe(II) atom is essential for the correct binding of the oxime via the N-atom of the oxime (Figure 2); Fe(III) is inactive.

Currently, there are about six characterized Oxds in bacteria (Table 1), and two in fungi [19,20]. The two Oxds in *Pseudomonas* are ≈90% identical [15,21]. Another pair of very similar Oxds (≈96% identity) is from *Rhododoccus* [22,23]. The Oxds of *Rhodococcus* are ≈76% identical to those of *Pseudomonas*, but those of *Bradyrhizobium* sp. [24] and *Bacillus* sp. [25] are more distant with ≈47% and ≈32% identity, respectively.

**Figure 3:**
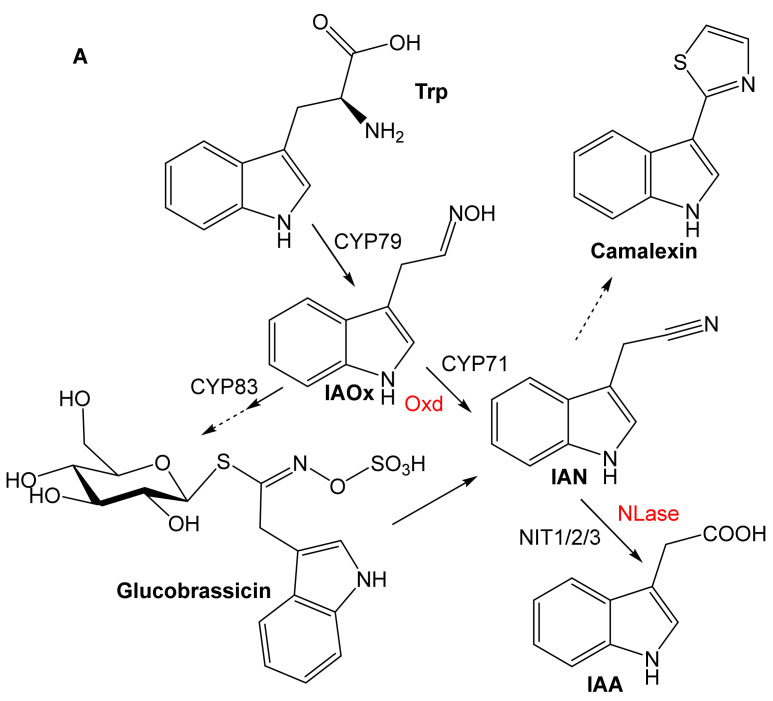

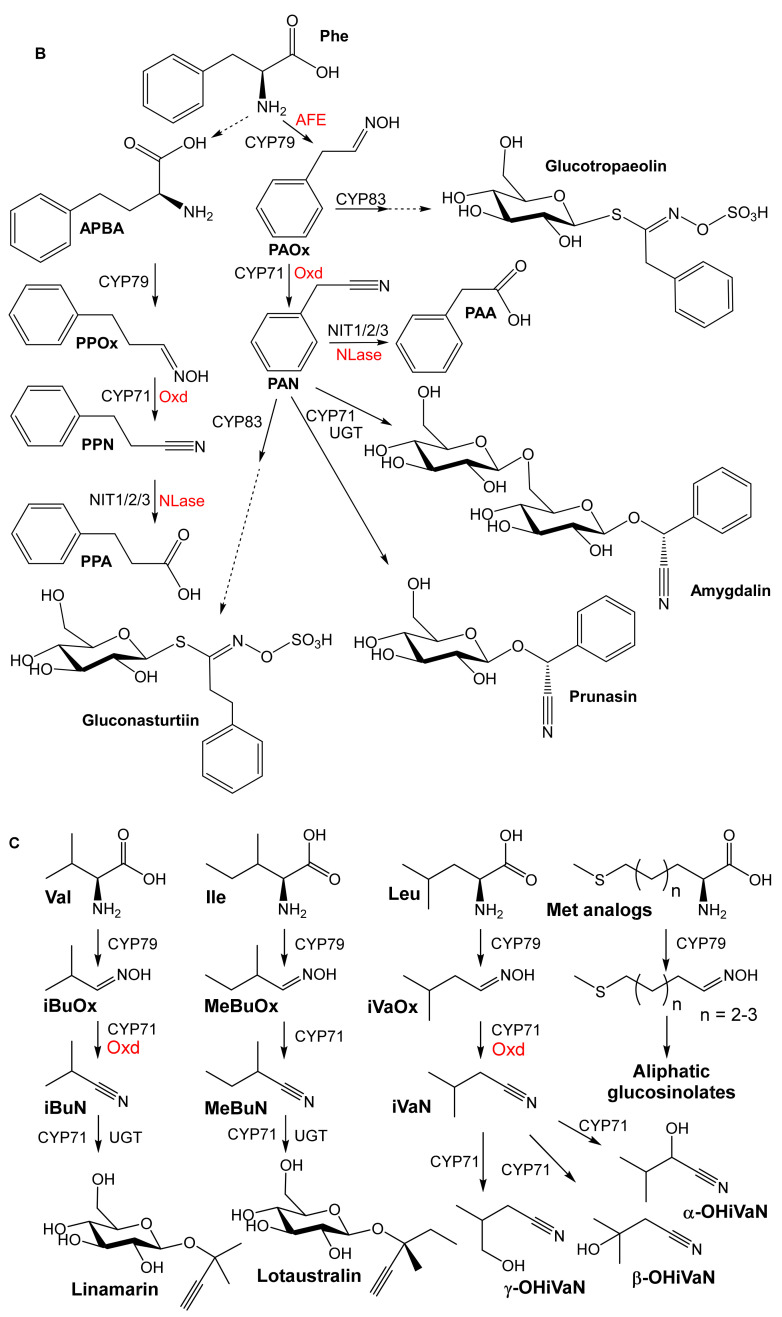
Plant and bacterial metabolism of (**A**) indole-3-acetaldoxime (IAOx), (**B**) phenylacetaldoxime (PAOx), 3-phenylpropionaldoxime (PPOx), (**C**) isobutyraldoxime (iBuOx), 2-methylbutyraldoxime (MeBuOx), isovaleraldoxime (3-methylbutyraldoxime; iVaOx), and methionine analogs (according to [1,3,27], modified). Plant and bacterial enzymes are shown in black and red, respectively. The hydroxy derivatives of iVaN are transformed to various glucosides (not shown). AFE, aldoxime-forming enzymes; APBA, 2-amino-4-phenylbutyric acid; CYP, cytochrome P450 (CYP71, CYP79, and CYP83 family); IAN, indole-3-acetonitrile; IAA, indole-3-acetic acid; iBuN, isobutyronitrile; iVaN, isovaleronitrile; MeBuN, 2-methylbutyronitrile; NIT1/2/3, plant nitrilases of NIT1, NIT2, and NIT3 type; NLase, nitrilase; PAA, phenylacetic acid; PAN, phenylacetonitrile; PPA, 3-phenylpropionic acid; PPN, 3-phenylpropionitrile; UGT, UDP-glucosyl transferase.

The characterized Oxds transform several plant aldoximes in addition to other (non-natural) aldoximes. The plant oximes were identified based on the above-mentioned review [1]. They are IAOx, phenylacetaldoxime (PAOx), 3-phenylpropionaldoxime (PPOx), isobutyraldoxime (iBuOx), and isovaleraldoxime (iVaOx). IAOx is formed from l-Trp and is a precursor of glucobrassicin and camalexin that is formed via IAN (Figure 3A). However, its role as a precursor of IAA is not entirely clear (Section 4). PAOx is formed from l-Phe, and its dehydration yields phenylacetonitrile (PAN). It is also a precursor of amygdalin or prunasin (CGs) (Figure 3B).

PPOx is formed from l-Phe via 2-amino-4-phenylbutyric acid (APBA) and is a precursor of gluconasturtiin (Figure 3B). The aliphatic oximes isobutyraldoxime (iBuOx) and isovaleraldoxime (iVaOx) are synthesized from l-Val and l-Leu, respectively (Figure 3C). They are widespread anti-herbivore compounds and floral scents. In addition, iBuOx is a precursor of linamarin [1], whereas the transformation of iVaOx leads to *α*-, *β*-, and *γ*-hydroxynitriles—precursors of the glucosides epidermin, epiheterodendrin, (dihydro)osmaronin, and sutherlandin [27]. Met analogs are transformed to thioaldoximes, which are precursors of aliphatic glucosinolates in Brassicales (*Arabidopsis*) [1]. Bacterial enzymes that may transform the plant compounds are also shown in Figure 3.

OxdB of *Bacillus* sp. OxB-1 [14], OxdK of *Pseudomonas* sp. K-9 [21], and OxdRG of *Rhodococcus globerulus* A-4 [22] transformed IAOx, PAOx, PPOx, and at least one aliphatic oxime (Table 2). However, their preferred substrates are different: arylaliphatic aldoximes (PPOx, PAOx) in OxdB and OxdRG and the aliphatic compound iVaOx in OxdK. IAOx is an acceptable substrate for OxdB and OxdRG, but OxdK has a very low activity for it. The *K*_M_ values are relatively high (≈0.5−5.5 mM) in all cases.

OxdRG showed lower *V*_max_ values [22] than the other Oxds. However, the activity increased under altered conditions [21]. In particular, anaerobic conditions were important to prevent the oxidation of the Fe(II) cofactor. Thus, the specific activity increased from less than 0.1 U/mg to 633 U/mg for *Z*-PAOx [21]. However, kinetic data were not reported for these conditions.

OxdBr1 of *Bradyrhizobium* sp. LTSPM299 was found to be similar to OxdB and OxdRG in terms of substrate specificity [24]. The Oxd of *Rhododoccus* sp. YH3-3 was tested with whole cells. iBuOx was dehydrated, but not IAOx, PAOx, or PPOx [28]. Kinetic data were not determined for these enzymes.

### 2.2. Nitrile-Converting Enzymes

**Figure 4:**
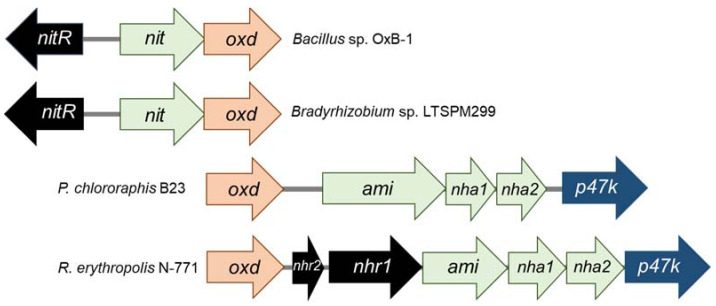
Genes encoding enzymes of the aldoxime-nitrile pathway form clusters (according to [15,21,23,24], modified). Examples: *Bacillus* sp. OxB-1, *Bradyrhizobium* sp. LTSPM299, *Pseudomonas chlororaphis* B23, and *Rhodococcus erythropolis* N-771. Encoded enzymes: *nit*, NLase; *nha1*, NHase α-subunit; *nha2*, NHase β-subunit. Blue arrows indicate genes coding for the putative NHase activator P47K. Black arrows indicate genes coding for putative transcriptional regulators (type specified). The flanking regions in *P. chlororaphis* and *R. erythropolis* contain some unidentified genes (not shown).

The above Oxd-producing bacteria also harbor genes for nitrile-converting enzymes—NLases in *Bacillus* sp. and *Bradyrhizobium* sp. and NHases in *P. chlororaphis* and *Rhodococcus erythropolis*. The genes for Oxd and NLase or NHase/Ami form clusters that are species- and strain-specific (Figure 4).

Two hypothetical genes were found upstream of the *oxd* gene in *Bacillus* and *Brady-rhizobium* [23,24]. They probably encode an NLase and a transcriptional regulator NitR, which belongs to the AraC family. The clusters in *Rhodococcus* and *Pseudomonas* are more complex. Both encode two NHase subunits, an amidase, and the NHase activator P47K [14,15]. The NHases in *P. chlororaphis* B23 and *R. erythropolis* N-771 are of Fe-type (with an Fe(III) cofactor) [29,30]. The NHase activator likely incorporates Fe into the active site [31]. However, its homolog in *Pseudomonas* sp. was found to be not necessary for NHase activity [32].

The substrate specificities of the NHases linked to Oxds are partially known. The NHase of *P. chlororaphis* transforms *n*-alkyl nitriles but not iVaN or PAN [29]. In contrast, the NHase from *R. erythropolis* N-771 was found to have a good activity for iBuN [30]. The NHase in *Rhododoccus* sp. YH3-3 was also characterized [28]. It belongs to the Co-type (with a Co(III) cofactor). It showed high activity for PAN, but lower activities for iBuN, IAN, and 3-phenylpropionitrile (PPN). Thus, some of the nitriles produced by the Oxds are substrates of NHases.

In contrast, less is known about the substrate specificity of the above NLases. The NLase of *Bacillus* sp. hydrolyzed PAN [14]. The NLase of *Bradyrhizobium* sp. LTSPM299 was not studied but it has a close homolog with activity for mandelonitrile [24]. This homolog was expressed from metagenomic DNA [33]. In addition, another homolog was characterized in *Bradyrhizobium japonicum* USDA 110 [34], which was reclassified as *Bradyrhizobium diazoefficiens* [26]. The best substrates of this homolog were found to be mandelonitrile and PAN [34].

## 3. Taxonomic and Ecological Distribution

The distribution of Oxds in bacteria was assessed based on activity and gene screening [35,36] and on data from the GenBank database [26] (Table 3).

The screening of Oxd activities included 540 bacterial strains and 435 fungal strains including yeasts [35]. The substrates tested were PAOx and pyridine-3-aldoxime. The latter was not one of the plant oximes [1]. Therefore, we focus on the activity for PAOx. This was present at significant levels in over twenty bacteria and over thirty fungi including two yeasts. The study also examined the hydrolysis of the corresponding nitriles. These activities were generally present in the strains with Oxd activities. The Oxd activities were most abundant in rhodococci.

In addition, the distribution of Oxds in some bacteria selected by the above screening was investigated by Southern hybridization and PCR amplification [36]. The hybridization procedure used probes based on *oxd* genes from *R. erythropolis* N-771. This allowed for the identification of *oxd* genes in *Rhodococcus* sp. NCIBM 11215 and NCIBM 11216, *R. erythropolis* strains JCM 3201, BG13, and B16, *Brevibacterium butanicum* ATCC 21196, and *Pseudomonas* sp. K-9. These strains and *Bacillus* sp. OxB-1 also gave positive results in PCR amplification with primers based on conserved *oxd* regions. PCR was also performed with primers for Fe-type and Co-type NHases. The *nha1* genes (Figure 4) were found in almost all strains of rhodococci shown to have *oxd* genes, in *Pseudomonas* sp. K-9, and in *B. butanicum* ATCC 21196. All of these NHases were identified as Fe-type.

According to GenBank searches, Oxds are most abundant in the phyla Actinobacteria and Proteobacteria. The phyla Firmicutes and Bacteroidetes contain few Oxds.

Among the Actinobacteria, the greatest number of putative Oxds was found in *Rhodococcus*, in accordance with the above-mentioned activity screening. A few were also found in *Arthrobacter*, *Corynebacterium*, *Microbacterium,* or *Streptomyces*. These bacteria are widely distributed in water and soils, typically in the rhizosphere [37,38,39]. They also include endophytic bacteria belonging to *Rhododoccus* [40] or *Streptomyces* [41].

Among the Proteobacteria, Oxds are most abundant in the *Bradyrhizobium*, *Variovorax,* and *Pseudomonas* genera. The habitat of *Bradyrhizobium* is typically the rhizosphere but also forest soils [43,44]. Members of the genus *Pseudomonas* occur, e.g., in soils and the rhizosphere [39,45] or as endophytes [45]. *Variovorax* is common in the rhizosphere among several other environments [46]. Several Oxds also occur in each of the genera *Rhizobium* (rhizobacteria [47]) and *Agrobacterium* (endophytes, rhizobacteria; [37,48,49]).

In the Firmicutes, most of the Oxds were found in *Bacillus*, *Brevibacillus* and the related genus *Siminovitchia*, while some other genera contain a single Oxd. Bacilli have a number of different habitats including the rhizosphere and also include endophytic bacteria [45].

In general, the genera with *oxd* genes also contain nitrilase (*nit*) genes. NLases are much more diverse than Oxds in terms of substrate specificity. There are several types of NLases: aromatic NLases, arylacetoNLases, plant NLase homologs, thermostable NLase, etc. [2,50]. In addition, some putative NLases differ significantly in sequence from characterized NLases, and thus may have other substrate specificities. NLases are distributed throughout bacterial phyla. The highest number of NLase sequences was found in *Rhododoccus*, *Streptomyces*, *Paraburkholderia*, *Pseudomonas*, *Rhizobium*, *Variovorax,* and *Flavobacterium*. The typical habitats of these bacteria are soils.

NHases are more conserved; Fe-type and Co-type NHases are about 50% identical. They are less widely distributed than NLases, as only a few were found in Firmicutes and Bacteroidetes. In Actinobacteria and Proteobacteria, their distribution is similar to that of NLases, with a few exceptions.

## 4. Potentiality of the Aldoxime-Nitrile Pathway in Plant-Associated Bacteria

The bacterial ability to detoxify oximes and nitriles appears to be advantageous in both mutualistic and parasitic lifestyles. Bacterial symbionts may also benefit from this ability in different ways, such as modulating plant growth through interactions with plant hormone synthesis.

The interaction of plant-growth-promoting bacteria with the host plant is complex. The beneficial effects are associated with the bacterial ability to produce IAA, nutrients (ammonia, phosphate), surfactants, siderophores, or enzymes that attack the cell wall polysaccharides in fungal pathogens [51]. Other mechanisms strengthen the plant cell wall, prevent an excessive production of the plant hormone ethylene [51], or change the distribution of the plant hormone abscisic acid [52].

**Figure 5:**
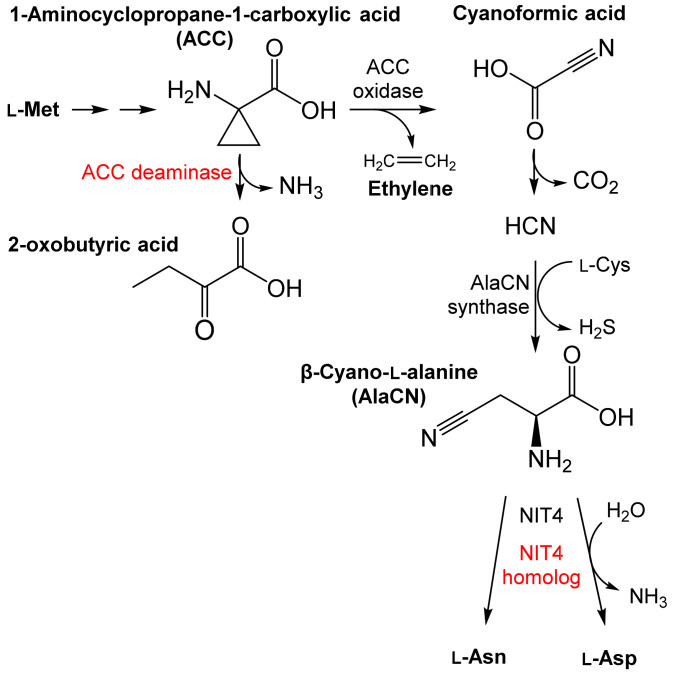
Biosynthesis of phytohormone ethylene in plants and detoxification of the byproduct HCN via β-cyano-l-alanine (AlaCN) (according to [2,52], modified). The synthesis of AlaCN proceeds in plants and, rarely, in bacteria. The hydrolysis of AlaCN proceeds in plants but also in some bacteria and fungi. NIT4, nitrilase specific for AlaCN (plant enzymes in black, bacterial enzymes in red).

In the pathway to ethylene (Figure 5), the intermediate 1-aminocyclopropane-1-carboxylate (ACC) can be degraded by the bacterial ACC deaminase [52] (EC 3.5.99.7). If not degraded, ACC is transformed to ethylene and cyanoformic acid by the plant ACC oxidase (EC 1.14.17.4); cyanoformic acid decomposes to CO_2_ and HCN. The latter is eliminated in the plant by reaction with L-Cys to form β-cyano-L-alanine (AlaCN). The enzyme that catalyzes this reaction (AlaCN synthase, EC 4.4.1.9) is widespread in plants but has been found in few bacteria [53]. The next step is the hydrolysis of AlaCN [2]. Plant NLases of the NIT4 type are highly specific for AlaCN [2,3]. The presence of similar NLases in bacteria such as *Pseudomonas fluorescens* SBW25 [2,54] and fungi [55] suggests the possibility of horizontal gene transfer (HGT). HGT from bacteria to fungi was already demonstrated for ACC deaminase (*acdS*) genes [56]. The substrate specificity distinguishes NIT4 from NLases typical of the aldoxime–nitrile pathway.

**Figure 6:**
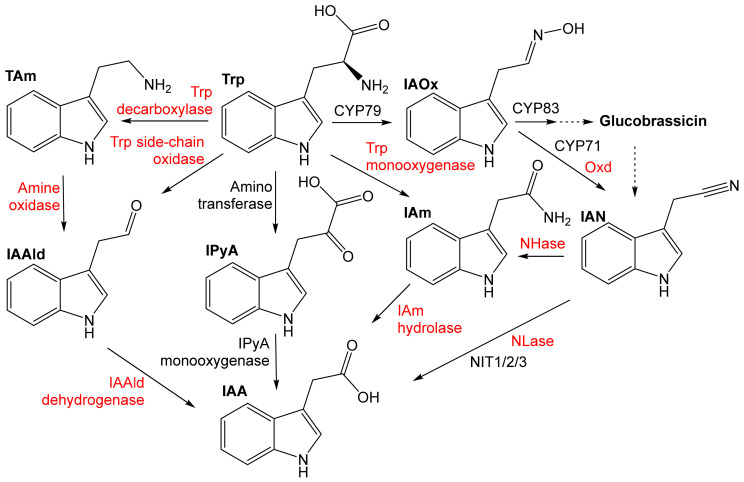
Trp-dependent biosynthesis of indole-3-acetic acid (IAA) in bacteria and plants (according to [1,3,10,57,58,59], modified). Enzymes typical of plants and bacteria are shown in black and red, respectively. IAA, indole-3-acetic acid; IAAld, indole-3-acetaldehyde; IAOx, indole-3-acetaldoxime; IAm, indole-3-acetamide; IAN, indole-3-acetonitrile; CYP, cytochrome P450 (CYP71, CYP79, and CYP83 family); IPyA, indole-3-pyruvate; NIT1/2/3, plant nitrilases of NIT1, NIT2, and NIT3 type; NLase, nitrilase.

The Trp-dependent pathways to the phytohormone IAA involve the indole-3-acetamide (IAm), indole-3-pyruvate (IPyA), tryptamine (TAm), Trp side-chain oxidase (TSO), and IAN pathway (Figure 6). The preferential pathways in plants and microbes are different. Plants have been assumed to mainly synthesize IAA via IPyA, tryptamine, and probably also IAM and IAN. Currently, the most studied metabolic pathway is the Trp–IPyA–IAA pathway catalyzed by an amino transferase and a monooxygenase of the YUCCA family [57,58]. IAAld has been found not to be an intermediate of the IPyA pathway, as previously thought [59]. Bacteria have been assumed to largely produce IAA via the IAM and IPyA pathway, with some rare examples of the IAN, TAM, and TSO pathway [10]. It is still largely unclear to what extent IAA is formed in bacteria or plants from IAOx or IAN. Plants probably hydrolyze IAN to IAA under certain conditions (lack of sulfur, infection) [3]. Both bacteria and plants may also synthesize IAA by the Trp-independent pathways from indole or indole-3-glycerolphosphate [10].

IAA has versatile biological activities. It supports germination and growth, triggers plant resistance mechanisms, and also has nematocidal activity [60]. However, IAA only has plant-promoting effects within a restricted range of concentrations [51]. The regulation of IAA production in bacteria is also sensitive to environmental factors [10]. The production of IAA by microbes can interfere with plant defense and physiology in many ways.

The endophyte *P. fluorescens* BRZ63 showed biocontrol activity against some fungal pathogens (*Rhizoctonia*, *Sclerotinia*, *Colletotrichum*, and *Fusarium*) in *Brassica napus* (oilseed rape). The bacterium improved plant fitness by a complex mechanism that also included the production of significant concentrations of IAA (0.06 mg/mL) [51]. Another strain of *P. fluorescens* protected the host plant (*Medicago trunculata*, a legume) against the fungus *Botrytis cinerea* at lower IAA concentrations (0.022 mg/mL). The strains with lower IAA production (<0.001 to ≈0.01 mg) did not promote shoot growth, notwithstanding the fungal infection [61].

Production of IAA from IAN or IAOx in *P. fluorescens* is possible, as the corresponding genes are present. In addition, IAN can be produced from glucobrassisin in *Brassicaceae* [3]. On the other hand, *P. fluorescens* may also transform Trp to IAA via IAAld produced by direct oxidation of Trp under specific conditions (stationary phase) [10].

*P. fluorescens* has several tens of putative Oxds. Some of them are most similar to OxdK from *Pseudomonas* sp. K-9 (up to 97% identity) and some to OxdRG from *R. globerulus* A-4 (up to 78% identity). OxdK has a non-zero activity for IAOx, and OxdRG has a good activity for this substrate (Section 2). *P. fluorescens* also has dozens of NLases. One of them (AAW79573) is an arylacetoNLase with significant activities for PAN, IAN, or PPN, and also for mandelonitrile. In this strain, *P. fluorescens* EBC191, genes corresponding to the mandelate pathway were found in the proximity of the *nit* gene but no *oxd* gene was found [62]. Thus, it is an example of an NLase that is not part of the ANP. However, a putative indole-3-acetamide (IAm) hydrolase gene was present upstream of the *nit* gene. IAm hydrolase is an enzyme of the IAm pathway [10] (Figure 6). Functional expression of the genes for the mandelate pathway was confirmed by demonstrating the growth of the strain on mandelate as the sole source of N; this pathway, together with nitrilase, is thought to allow for bacterial utilization of mandelonitrile released from plants [62].

The rhizobacterium *Variovorax paradoxus* 5C-2 supports plant growth in a variety of ways, including ACC deamination (Figure 5) and abscisic acid distribution between plant tissues in *Pisum sativum* [52]. It also produces IAA (e.g., 0.075 mg/L [52]) in media containing Trp [52,63]. However, Trp concentrations in plant exudates (the Trp source for rhizobacteria) may not be sufficient [64]. This allows us to consider alternative pathways of IAA synthesis such as the aforementioned Trp-independent pathway [10]. However, we also cannot exclude the synthesis of IAA from the plant-produced IAOx or IAN. *V. paradoxus* has 12 putative Oxds, all of which are relatively similar (≈50% identical) to the Oxds active for IAOx (OxdBr1, OxdRG). The species *V. paradoxus* also contains over 40 NLases. Most of them are closely related to the NLase Nit6803 from *Synechocystis* sp. PCC6803 [65], whose homologs are collectively referred to as the Nit6803 NLase family. The best substrate of this NLase is fumaronitrile, but a variety of structurally diverse substrates including IAN are also accepted [66].

Fungi are also able to transform oximes to carboxylic acids, which may add complexity to plant-microbial interactions. One of the oilseed rape (*Brassica napus*) pathogens, *Sclerotinia sclerotiorum* clone #33 [51], has an Oxd with a narrow specificity for a few aldoximes, while IAOx is its best substrate. However, the activity of the purified enzyme is low (0.014 U/mg protein) [19]. Another Oxd was characterized in *Fusarium graminearum* MAFF305135, which causes head blight in some crops. This enzyme showed high activity for IAOx (*V*_max_ of 19.3 U/mg protein), but also for PAOx, PPOx, and aliphatic nitriles [20]. These are the only two fungal Oxds that have been characterized, but Ascomycota contain many more Oxds. Hundreds of fungal Oxd sequences have been deposited in GenBank, while less than ten were found in Basidiomycota. Ascomycota are also remarkably rich in NLases with several thousands of sequences, while approximately two hundred NLases were found in Basidiomycota [55,67]. *Fusarium* and its teleomorph *Gibberella* have dozens of NLases, some of which were characterized. They include several substrate-specificity types [6]. They are generally active towards PAN, whereas their specificity for other natural nitriles is still largely unclear.

The transformation of mutualistic bacteria to pathogens is relatively simple. For example, the pathogenicity of *Rhodococcus fascians* D188 depends on the production of cytokinins. This was enabled in the strains that had acquired the virulent plasmid [68,69]. *R. fascians* increases the IAA concentration in the plant by about tenfold, but the role of this effect in pathogenesis is largely unclear. No intermediates corresponding to the IAN, IAM, or TAm pathways were found at significant levels. The study suggested that the indole-3-pyruvate pathway (Figure 6) is the main route to IAA in this organism [70]. Thus, the role of the approximately ten *oxd* genes found in this taxon is still poorly understood.

## 5. Conclusions

The long coexistence of plants and microbes has led to a variety of close relationships (symbiosis). The bacterial symbionts must have pathways that detoxify plant defenses. One of these pathways is ANP, which likely confers detoxification and assimilation capabilities to the bacteria. It may also have a signaling function. Here, we summarized the data that shed light on the role of this pathway in plant-associated bacteria and the interaction with their host plants. Moreover, NLases are much more diverse than Oxds in terms of sequence and substrate specificity. They can also act outside the ANP, e.g., in the degradation of the toxic plant metabolites AlaCN and mandelonitrile.

First, we analyzed data on the substrate specificity of the characterized bacterial Oxds. It has been shown that some plant oximes are Oxd substrates in vitro. We have also investigated whether the nitrile-converting enzymes in the same organisms are capable of converting the Oxd products—nitriles. This is likely for most nitriles, as indicated by the data on these enzymes or their homologs.

We then examined the distribution of Oxds using data from activity screening and database mining. It became clear that there are hundreds of *oxd* genes in bacteria, but they are unevenly distributed. Oxds are only abundant in four genera—*Bradyrhizobium*, *Rhodoccus*, *Pseudomonas*, and *Variovorax*—along with NLases or NHases. NLases are the most abundant. Oxds and nitrile-converting enzymes are common in both plant-growth-promoting bacteria and plant pathogens, and can alter plant growth or defense via, e.g., IAA production. However, studies that could explain the mechanisms of this plant-bacterial cross-talk are largely lacking.

## Figures and Tables

**Table 1:** Identities (approximated) between amino acid sequences of bacterial aldoxime dehydratases (according to [26]). Coverage % in brackets.

	OxdB (BAA90461.1)	OxdRG (BAC99076.1) ^1^	OxdK (BAD98528.1) ^2^	OxdBr1 (WP_044589203)
	(*Bacillus* sp. OxB-1) [25]	(*Rhododoccus globerulus* A-4 [22]	(*Pseudomonas* sp. K-9) [21]	(*Bradyrhizobium* sp. LTSPM299) [24]
OxdB	−	32 (92)	32 (93)	32 (93)
OxdRG	−	−	76 (99)	47 (98)
OxdK	−	−	−	47 (99)

^1^ 96% identity (100% coverage) to Oxd in *Rhododoccus erythropolis* N-771 (3A15_A) [23]. ^2^ 90% identity (100% coverage) to Oxd in *Pseudomonas chlororaphis* B23 (3W08_A) [15].

**Table 2:** Substrate specificities of selected bacterial aldoxime dehydratases toward plant oximes.

Enzyme ^1^	Substrate ^2^	Relative Activity (%)	*V*_max_ [U/mg]	*K*_M_ [mM]	*V*_max_/*K*_M_ [U/mg/mM]
OxdB [14]	IAOx	18	5.42	2.40	2.26
	iVaOx	20	7.72	3.58	2.16
	***Z*-PAOx**	**100**	**19.5**	**0.872**	**22.4**
	*Z*-PPOx	63	14.3	1.36	10.5
OxdK [21]	IAOx	1	n.d.	n.d.	n.d.
	iBuOx	14	5.87	0.538	10.9
	**iVaOx**	**100**	**35.1**	**1.33**	**26.4**
	*Z*-PAOx	7	2.61	0.991	2.63
	*Z*-PPOx	31	12.1	0.975	12.4
OxdRG [22]	IAOx	11	0.281	3.91	0.072
	iBuOx	39	0.041	5.54	0.007
	iVaOx	87	0.239	3.97	0.060
	*Z*-PAOx	40	0.140	1.40	0.100
	***Z*-PPOx**	**100**	**0.392**	**2.31**	**0.170**

^1^ See Table 1. ^2^ IAOx, indole-3-acetaldoxime; iBuOx, isobutyraldoxime; iVaOx, isovaleraldoxime (3-methylbutyraldoxime); PAOx, phenylacetaldoxime; PPOx, 3-phenylpropionaldoxime. The substrates are of *E*/*Z*-configuration unless noted otherwise. Substrates with the highest relative activity and *V*_max_/*K*_M_ are in bold.

**Table 3:** Distribution of aldoxime dehydratases, nitrilases, and nitrile hydratases in bacteria.

Phylum	Genus	Number of Sequences ^1^			Number of Active Strains ^2^
		Oxd	NLase	NHase	
Actinobacteria	*Arthrobacter*	5	16	5	n.d.
	*Cellulomonas*	-	10	-	n.d.
	*Corynebacterium*	3	3	-	2
	*Brevibacterium*	1	41	1	n.d.
	*Gordonia*	4	6	13	n.d.
	*Microbacterium*	5	27	9	1
	*Nitriliruptor*	2	3	1	n.a.
	*Micrococcus*	-	-	-	1
	*Nocardia*	1	36	7	2
	*Rhodococcus*	142	190	107	14
	*Streptomyces*	8	157	252	n.d.
Firmicutes	*Bacillus*	2	15	2	2
	*Brevibacillus*	2	66	-	n.a.
	*Cytobacillus*	1	1	-	n.a.
	*Geomicrobium*	1	1	-	n.a.
	*Paenibacillus*	1	99	13	n.a.
	*Siminovitchia*	3	14	-	n.a.
Proteobacteria	*Afipia*	2	-	14	n.a.
	*Agrobacterium*	9	99	43	n.d.
	*Bradyrhizobium*	248	101	298	n.a.
	*Erwinia*	1	19	5	n.d.
	*Gibbsiella*	1	10	2	n.a.
	*Herbaspirillum*	1	45	9	n.a.
	*Klebsiella*	-	17	66	n.d.
	*Novosphingobium*	1	68	1	n.a.
	*Paraburkholderia*	1	336	139	n.a.
	*Proteus*	-	1	2	n.d.
	*Pseudomonas*	48	261	50	2
	*Pseudomaricurvus*	1	-	-	n.a.
	*Rhizobium*	7	270	600	n.a.
	*Serratia*	-	4	11	n.d.
	*Variovorax*	73	156	92	n.d.
	*Xanthomonas*	2	1	2	n.d.
Bacteroidetes ^3^	*Flavobacterium*	-	172	4	n.d.
	*Maribacter*	1	58	-	n.a.

^1^ Retrieved from [26]. Searches for hypothetical Oxds were performed using OxdBr1 (WP_044589203 [24]) as a template. Searches for hypothetical NLases and NHases were performed using the NLase from *Bradyrhizobium diazoefficiens* (NP_770037 [34]) and the α-subunit of the NHase from *Rhododoccus erythropolis* N-771 (2AHJ_A [42]) as templates, respectively. ^2^ According to [35]. The active strains exhibited both aldoxime dehydratase and nitrile-hydrolyzing activity for phenylacetaldoxime and phenylacetonitrile, respectively; n.a., not assayed; n.d., not detected. ^3^ Other four Oxds were identified in a bacterium of the *Chitinophagaceae* family in Bacteroidetes.

## References

[1] Sørensen M., Neilson E.H.J., Møller B.L. (2018). Oximes: Unrecognized chameleons in general and specialized plant metabolism. Mol. Plant.

[2] Howden A.J., Preston G.M. (2009). Nitrilase enzymes and their role in plant-microbe interactions. Microb. Biotechnol..

[3] Piotrowski M. (2008). Primary or secondary? Versatile nitrilases in plant metabolism. Phytochemistry.

[4] Kato Y., Tsuda T., Asano Y. (2007). Purification and partial characterization of *N*-hydroxy-l-phenylalanine decarboxylase/oxidase from *Bacillus* sp. strain OxB-1, an enzyme involved in aldoxime biosynthesis in the “aldoxime-nitrile pathway”. Biochim. Biophys. Acta.

[5] Betke T., Higuchi J., Rommelmann P., Oike K., Nomura T., Kato Y., Asano Y., Gröger H. (2018). Biocatalytic synthesis of nitriles through dehydration of aldoximes: The substrate scope of aldoxime dehydratases. ChemBioChem.

[6] Martínková L. (2019). Nitrile metabolism in fungi: A review of its key enzymes nitrilases with focus on their biotechnological impact. Fungal Biol. Rev..

[7] Shen J.-D., Cai X., Liu Z.-Q., Zheng Y.-G. (2020). Nitrilase: A promising biocatalyst in industrial applications for green chemistry. Crit. Rev. Biotechnol..

[8] Stolz A., Eppinger E., Sosedov O., Kiziak C. (2019). Comparative analysis of the conversion of mandelonitrile and 2-phenylpropionitrile by a large set of variants generated from a nitrilase originating from *Pseudomonas fluorescens* EBC191. Molecules.

[9] Bhalla T.C., Kumar V., Kumar V. (2018). Enzymes of aldoxime-nitrile pathway for organic synthesis. Rev. Environ. Sci. Bio-Technol..

[10] Spaepen S., Vanderleyden J., Remans R. (2007). Indole-3-acetic acid in microbial and microorganism-plant signaling. FEMS Microbiol. Rev..

[11] Santoyo G., Moreno-Hagelsieb G., Orozco-Mosqueda Mdel C., Glick B.R. (2016). Plant growth-promoting bacterial endophytes. Microbiol. Res..

[12] Mahadevan S. (1963). Conversion of 3-indolacetaldoxime to 3-indoleacetonitrile by plants. Arch. Biochem. Biophys..

[13] Kato Y., Ooi R., Asano Y. (1998). Isolation and characterization of a bacterium possessing a novel aldoxime-dehydration activity and nitrile-degrading enzymes. Arch. Microbiol..

[14] Kato Y., Nakamura K., Sakiyama H., Mayhew S.G., Asano Y. (2000). Novel heme-containing lyase, phenylacetaldoxime dehydratase from *Bacillus* sp. strain OxB-1: Purification, characterization, and molecular cloning of the gene. Biochemistry.

[15] Oinuma K., Hashimoto Y., Konishi K., Goda M., Noguchi T., Higashibata H., Kobayashi M. (2003). Novel aldoxime dehydratase involved in carbon-nitrogen triple bond synthesis of *Pseudomonas chlororaphis* B23. Sequencing, gene expression, purification, and characterization. J. Biol. Chem..

[16] Martínková L., Rucká L., Nešvera J., Pátek M. (2017). Recent advances and challenges in the heterologous production of microbial nitrilases for biocatalytic applications. World J. Microbiol. Biotechnol..

[17] Asano Y., Tani Y., Yamada H. (1980). A new enzyme nitrile hydratase which degrades acetonitrile in combination with amidase. Agr. Biol. Chem..

[18] Hinzmann A., Betke T., Asano Y., Gröger H. (2021). Synthetic processes toward nitriles without the use of cyanide: A biocatalytic concept based on dehydration of aldoximes in water. Chem. Eur. J..

[19] Pedras M.S., Minic Z., Thongbam P.D., Bhaskar V., Montaut S. (2010). Indolyl-3-acetaldoxime dehydratase from the phytopathogenic fungus *Sclerotinia sclerotiorum*: Purification, characterization, and substrate specificity. Phytochemistry.

[20] Kato Y., Asano Y. (2005). Purification and characterization of aldoxime dehydratase of the head blight fungus, *Fusarium graminearum*. Biosci. Biotechnol. Biochem..

[21] Kato Y., Asano Y. (2006). Molecular and enzymatic analysis of the “aldoxime-nitrile pathway” in the glutaronitrile degrader *Pseudomonas* sp. K-9. Appl. Microbiol. Biotechnol..

[22] Xie S.X., Kato Y., Komeda H., Yoshida S., Asano Y. (2003). A gene cluster responsible for alkylaldoxime metabolism coexisting with nitrile hydratase and amidase in *Rhodococcus globerulus* A-4. Biochemistry.

[23] Kato Y., Yoshida S., Xie S.-X., Asano Y. (2004). Aldoxime dehydratase co-existing with nitrile hydratase and amidase in the iron-type nitrile hydratase-producer *Rhodococcus* sp. N-771. J. Biosci. Bioeng..

[24] Rädisch R., Chmátal M., Rucká L., Novotný P., Petrásková L., Halada P., Kotík M., Pátek M., Martínková L. (2018). Overproduction and characterization of the first enzyme of a new aldoxime dehydratase family in *Bradyrhizobium* sp.. Int. J. Biol. Macromol..

[25] Kato Y., Asano Y. (2003). High-level expression of a novel FMN-dependent heme-containing lyase, phenylacetaldoxime dehydratase of Bacillus sp. strain OxB-1, in heterologous hosts. Protein Expres. Purif..

[26] Basic Local Alignment Search Tool. https://blast.ncbi.nlm.nih.gov.

[27] Knoch E., Motawie M.S., Olsen C.E., Møller B.L., Lyngkjaer M.F. (2016). Biosynthesis of the leucine derived alpha-, beta- and gamma-hydroxynitrile glucosides in barley (*Hordeum vulgare* L.). Plant. J..

[28] Kato Y., Tsuda T., Asano Y. (1999). Nitrile hydratase involved in aldoxime metabolism from *Rhodococcus* sp. strain YH3-3 purification and characterization. Eur. J. Biochem..

[29] Nagasawa T., Nanba H., Ryuno K., Takeuchi K., Yamada H. (1987). Nitrile hydratase of *Pseudomonas chlororaphis* B23. Eur. J. Biochem..

[30] Nagamune T., Kurata H., Hirata M., Honda J., Koike H., Ikeuchi M., Inoue Y., Hirata A., Endo I. (1990). Purification of inactivated photoresponsive nitrile hydratase. Biochem. Biophys. Res. Commun..

[31] Nojiri M., Nakayama H., Odaka M., Yohda M., Takio K., Endo I. (2000). Cobalt-substituted Fe-type nitrile hydratase of *Rhodococcus* sp. N-771. FEBS Lett..

[32] Duca D., Rose D.R., Glick B.R. (2014). Characterization of a nitrilase and a nitrile hydratase from *Pseudomonas* sp. strain UW4 that converts indole-3-acetonitrile to indole-3-acetic acid. Appl. Environ. Microbiol..

[33] Robertson D.E., Chaplin J.A., DeSantis G., Podar M., Madden M., Chi E., Richardson T., Milan A., Miller M., Weiner D.P. (2004). Exploring nitrilase sequence space for enantioselective catalysis. Appl. Environ. Microbiol..

[34] Zhu D., Mukherjee C., Yang Y., Rios B.E., Gallagher D.T., Smith N.N., Biehl E.R., Hua L. (2008). A new nitrilase from *Bradyrhizobium japonicum* USDA 110. Gene cloning, biochemical characterization and substrate specificity. J. Biotechnol..

[35] Kato Y., Ooi R., Asano Y. (2000). Distribution of aldoxime dehydratase in microorganisms. Appl. Environ. Microbiol..

[36] Kato Y., Yoshida S., Asano Y. (2005). Polymerase chain reaction for identification of aldoxime dehydratase in aldoxime- or nitrile-degrading microorganisms. FEMS Microbiol. Lett..

[37] Sharma M., Mishra V., Rau N., Sharma R.S. (2016). Increased iron-stress resilience of maize through inoculation of siderophore-producing *Arthrobacter globiformis* from mine. J. Basic. Microbiol..

[38] Worsley S.F., Newitt J., Rassbach J., Batey S.F.D., Holmes N.A., Murrell J.C., Wilkinson B., Hutchings M.I. (2020). *Streptomyces* endophytes promote host health and enhance growth across plant species. Appl. Environ. Microbiol..

[39] Vick S.H.W., Fabian B.K., Dawson C.J., Foster C., Asher A., Hassan K.A., Midgley D.J., Paulsen I.T., Tetu S.G. (2021). Delving into defence: Identifying the *Pseudomonas protegens* Pf-5 gene suite involved in defence against secreted products of fungal, oomycete and bacterial rhizosphere competitors. Microb. Genom..

[40] Gonzalez-Benitez N., Martin-Rodriguez I., Cuesta I., Arrayas M., White J.F., Molina M.C. (2021). Endophytic microbes are tools to increase tolerance in *Jasione* plants against arsenic stress. Front. Microbiol..

[41] Yandigeri M.S., Meena K.K., Singh D., Malviya N., Singh D.P., Solanki M.K., Yadav A.K., Arora D.K. (2012). Drought-tolerant endophytic actinobacteria promote growth of wheat (*Triticum aestivum*) under water stress conditions. Plant Growth Regul..

[42] Song L., Wang M.Z., Shi J.J., Xue Z.Q., Wang M.X., Qian S.J. (2007). High resolution X-ray molecular structure of the nitrile hydratase from *Rhodococcus erythropolis* AJ270 reveals posttranslational oxidation of two cysteines into sulfinic acids and a novel biocatalytic nitrile hydration mechanism. Biochem. Biophys. Res. Commun..

[43] VanInsberghe D., Maas K.R., Cardenas E., Strachan C.R., Hallam S.J., Mohn W.W. (2015). Non-symbiotic *Bradyrhizobium* ecotypes dominate North American forest soils. ISME J..

[44] Jones F.P., Clark I.M., King R., Shaw L.J., Woodward M.J., Hirsch P.R. (2016). Novel European free-living, non-diazotrophic *Bradyrhizobium* isolates from contrasting soils that lack nodulation and nitrogen fixation genes—A genome comparison. Sci. Rep..

[45] Adeleke B.S., Babalola O.O., Glick B.R. (2021). Plant growth-promoting root-colonizing bacterial endophytes. Rhizosphere.

[46] Han J.I., Choi H.K., Lee S.W., Orwin P.M., Kim J., Laroe S.L., Kim T.G., O’Neil J., Leadbetter J.R., Lee S.Y. (2011). Complete genome sequence of the metabolically versatile plant growth-promoting endophyte *Variovorax paradoxus* S110. J. Bacteriol..

[47] Agrawal M., Archana G. (2021). Phenotypic display of plant growth-promoting traits in individual strains and multispecies consortia of plant growth promoting rhizobacteria and rhizobia under salinity stress. Rhizosphere.

[48] Guo H., Glaeser S.P., Alabid I., Imani J., Haghighi H., Kämpfer P., Kogel K.H. (2017). The abundance of endofungal bacterium *Rhizobium radiobacter* (syn. *Agrobacterium tumefaciens*) increases in its fungal host Piriformospora indica during the tripartite sebacinalean symbiosis with higher plants. Front. Microbiol..

[49] Cavalcanti M.I.P., Nascimento R.d.C., Rodrigues D.R., Escobar I.E.C., Fraiz A.C.R., de Souza A.P., de Freitas A.D.S., Nóbrega R.S.A., Fernandes-Júnior P.I. (2020). Maize growth and yield promoting endophytes isolated into a legume root nodule by a cross-over approach. Rhizosphere.

[50] Thuku R.N., Brady D., Benedik M.J., Sewell B.T. (2009). Microbial nitrilases: Versatile, spiral forming, industrial enzymes. J. Appl. Microbiol..

[51] Chlebek D., Pinski A., Żur J., Michalska J., Hupert-Kocurek K. (2020). Genome mining and evaluation of the biocontrol potential of *Pseudomonas fluorescens* BRZ63, a new endophyte of oilseed rape (*Brassica napus* L.) against fungal pathogens. Int. J. Mol. Sci..

[52] Jiang F., Chen L., Belimov A.A., Shaposhnikov A.I., Gong F., Meng X., Hartung W., Jeschke D.W., Davies W.J., Dodd I.C. (2012). Multiple impacts of the plant growth-promoting rhizobacterium *Variovorax paradoxus* 5C-2 on nutrient and ABA relations of *Pisum sativum*. J. Exp. Bot..

[53] Omura H., Kuroda M., Kobayashi M., Shimizu S., Yoshida T., Nagasawa T. (2003). Purification, characterization and gene cloning of thermostable *O*-acetyl-L-serine sulfhydrylase forming beta-cyano-L-alanine. J. Biosci. Bioeng..

[54] Howden A.J.M., Jill Harrison C., Preston G.M. (2009). A conserved mechanism for nitrile metabolism in bacteria and plants. Plant J..

[55] Rucká L., Kulik N., Novotný P., Sedova A., Petrásková L., Příhodová R., Křístková B., Halada P., Pátek M., Martínková L. (2020). Plant nitrilase homologues in fungi: Phylogenetic and functional analysis with focus on nitrilases in *Trametes versicolor* and *Agaricus bisporus*. Molecules.

[56] Bruto M., Prigent-Combaret C., Luis P., Moënne-Loccoz Y., Muller D. (2014). Frequent, independent transfers of a catabolic gene from bacteria to contrasted filamentous eukaryotes. Proc. R. Soc. B.

[57] Casanova-Sáez R., Voß U. (2019). Auxin metabolism controls developmental decisions in land plants. Trends Plant Sci..

[58] Enders T.A., Strader L.C. (2015). Auxin activity: Past, present, and future. Am. J. Bot..

[59] Mashiguchi K., Tanaka K., Sakai T., Sugawara S., Kawaide H., Natsume M., Hanada A., Yaeno T., Shirasu K., Yao H. (2011). The main auxin biosynthesis pathway in *Arabidopsis*. Proc. Natl. Acad. Sci. USA.

[60] Bogner C.W., Kamdem R.S., Sichtermann G., Matthäus C., Holscher D., Popp J., Proksch P., Grundler F.M., Schouten A. (2017). Bioactive secondary metabolites with multiple activities from a fungal endophyte. Microb. Biotechnol..

[61] Hernández-León R., Rojas-Solís D., Contreras-Pérez M., Orozco-Mosqueda M.d.C., Macías-Rodríguez L.I., Reyes-de la Cruz H., Valencia-Cantero E., Santoyo G. (2015). Characterization of the antifungal and plant growth-promoting effects of diffusible and volatile organic compounds produced by *Pseudomonas fluorescens* strains. Biol. Control.

[62] Kiziak C., Conradt D., Stolz A., Mattes R., Klein J. (2005). Nitrilase from *Pseudomonas fluorescens* EBC191: Cloning and heterologous expression of the gene and biochemical characterization of the recombinant enzyme. Microbiology.

[63] Abdellatif L., Ben-Mahmoud O.M., Yang C., Hanson K.G., Gan Y., Hamel C. (2016). The H_2_-oxidizing rhizobacteria associated with field-grown lentil promote the growth of lentil inoculated with hup+ *Rhizobium* through multiple modes of action. J. Plant Growth Regul..

[64] Zhao S., Zhou N., Zhao Z.Y., Zhang K., Wu G.H., Tian C.Y. (2016). Isolation of endophytic plant growth-promoting bacteria associated with the halophyte *Salicornia europaea* and evaluation of their promoting activity under salt stress. Curr. Microbiol..

[65] Zhang L.J., Yin B., Wang C., Jiang S.Q., Wang H.L., Yuan Y.A., Wei D.Z. (2014). Structural insights into enzymatic activity and substrate specificity determination by a single amino acid in nitrilase from *Syechocystis* sp. PCC6803. J. Struct. Biol..

[66] Heinemann U., Engels D., Bürger S., Kiziak C., Mattes R., Stolz A. (2003). Cloning of a nitrilase gene from the cyanobacterium *Synechocystis* sp. strain PCC6803 and heterologous expression and characterization of the encoded protein. Appl. Environ. Microbiol..

[67] Rucká L., Chmátal M., Kulik N., Petrásková L., Pelantová H., Novotný P., Příhodová R., Pátek M., Martínková L. (2019). Genetic and functional diversity of nitrilases in Agaricomycotina. Int. J. Mol. Sci..

[68] Savory E.A., Fuller S.L., Weisberg A.J., Thomas W.J., Gordon M.I., Stevens D.M., Creason A.L., Belcher M.S., Serdani M., Wiseman M.S. (2017). Evolutionary transitions between beneficial and phytopathogenic *Rhodococcus* challenge disease management. Elife.

[69] Savory E.A., Weisberg A.J., Stevens D.M., Creason A.L., Fuller S.L., Pearce E.M., Chang J.H. (2020). Phytopathogenic *Rhodococcus* have diverse plasmids with few conserved virulence functions. Front. Microbiol..

[70] Vandeputte O., Oden S., Mol A., Vereecke D., Goethals K., El Jaziri M., Prinsen E. (2005). Biosynthesis of auxin by the gram-positive phytopathogen *Rhodococcus fascians* is controlled by compounds specific to infected plant tissues. Appl. Environ. Microbiol..

